# Female Japanese quail visually differentiate testosterone-dependent male attractiveness for mating preferences

**DOI:** 10.1038/s41598-018-28368-z

**Published:** 2018-07-03

**Authors:** Gen Hiyama, Shusei Mizushima, Mei Matsuzaki, Yasuko Tobari, Jae-Hoon Choi, Takashi Ono, Masaoki Tsudzuki, Satoshi Makino, Gen Tamiya, Naoki Tsukahara, Shoei Sugita, Tomohiro Sasanami

**Affiliations:** 1 Department of Applied Biological Chemistry, Faculty of Agriculture, Shizuoka University, 836 Ohya, Shizuoka, Shizuoka, 422-8529 Japan; 2 Department of Biological Sciences, Faculty of Science, Hokkaido University, Kita 10 Nishi 8, Kita-ku, Sapporo, Hokkaido, 060-0810 Japan; 3 Laboratory of Animal Genetics and Breeding, School of Veterinary Medicine, Azabu University, Fuchinobe 1-17-71, Sagamihara, 252-5201 Japan; 4 Laboratory of Animal Breeding and Genetics, Graduate School of Biosphere Science, Hiroshima University, Higashi-Hiroshima, 739-8528 Japan; 5 Tohoku Medical Megabank Organization, Tohoku University, 2-1 Seiryo-machi, Aoba-ku, Sendai, Miyagi 980-8575 Japan; 6 RIKEN Center for Advanced Intelligence Project, 1-4-1 Nihonbashi, Chuo-ku, Tokyo 103-0027 Japan; 7 CrowLab Inc., Utsunomiya-ventures #3, Tochigi Prefecture Industrial Center, 3-1-4, Chuo, Utsunomiya-shi, Tochigi 320-0806 Japan; 8 Faculty of Agriculture, Utsunomiya University, Utsunomiya, Tochigi, 321-8505 Japan; 9 Present Address: Medical-Industrial Translational Research Center, Fukushima Medical University, 1 Hikarigaoka, Fukushima, 960-8031 Japan

## Abstract

Biased mating due to female preferences towards certain traits in males is a major mechanism driving sexual selection, and may constitute an important evolutionary force in organisms with sexual reproduction. In birds, although the role of male ornamentation, plumage coloration, genetic dissimilarity, and body size have on mate selection by females have been examined extensively, few studies have clarified exactly how these characteristics affect female mate preferences. Here, we show that testosterone (T)-dependent male attractiveness enhances female preference for males of a polygamous species, the Japanese quail. A significant positive correlation between female mating preference and circulating T in the male was observed. The cheek feathers of attractive males contained higher levels of melanin and were more brightly colored. The ability of females to distinguish attractive males from other males was negated when the light source was covered with a sharp cut filter (cutoff; < 640 nm). When females were maintained under short-day conditions, the expression of retinal red-sensitive opsin decreased dramatically and they became insensitive to male attractiveness. Our results showed that female preference in quail is strongly stimulated by male feather coloration in a T-dependent manner and that female birds develop a keen sense for this coloration due to upregulation of retinal red-sensitive opsin under breeding conditions.

## Introduction

Mating preferences leading to biased mating with the opposite sex is an important trait to produce descendants. Female mating preferences are affected by the trait expressed in male individuals, which in turn is often a proxy for male genetic quality^1–3^. Generally, males invest less energy than females in many reproductive events, such as egg production, gestation, delivery and nurturing. Consequently, males can often mate more frequently than females; male reproductive success is usually more variable and is dependent on access to multiple females with which to mate^4^. To increase mating opportunities, male animals have developed a variety of secondary sex characteristics. For instance, many insect species use sex pheromones to attract potential mating partners; for example, moths such as *Helicoverpa armigera*, employ specific chemicals to regulate mating and increase fecundity^5^. Female guppies (*Poecilia reticulata*) show a preference for males with large body sizes and more chromatic orange spots^6^. In medaka fish (*Oryzias latipes*), a similar preference was observed^7^, but social familiarity between males and females can also affect female mate preferences^8^.

It is known that visual sensitivity for color fluctuates seasonally. For instance, the evidence suggests that the expression levels of several kinds of retinal opsin genes in teleosts varied in response to photoperiod^9–11^. Even in humans, colour perception, especially for the color yellow changes between seasons^12^. Although these changes appear to cause alterations in the spectral sensitivity of vision in response to seasons, the physiological importance, as well as the underlying mechanism, remain unclear.

In birds, showy ornaments, plumage and courtship song/behavior in males are frequently employed to attract females. In studies on female mate choice, females often prefer more elaborate ornaments or showy plumage because these traits represent the heritable aspect of male quality, *i*.*e*., the well-ornamented males in the population are more likely to produce superior offspring (*i*.*e*. good gene model^13^). On the other hand, in the case of house finches (*Haemorhous mexicanus*), male plumage coloration is affected not only by heritability but also by diet, especially diet containing carotenoids, and females prefer males that have had their plumage experimentally brightened using hair dye^14^. It is considered that vibrantly colored feathers in male may be an indicator of good health, and thus being better able to obtain food. Consequently, female mate preference is biased towards healthy looking males. For example, roosters infected with parasites were not preferred by females^15^. Thus, mate selection based on male characteristics is linked to the heritable aspect of male quality, foraging ability and parasite resistance all of which may have the effect of increasing reproductive success in females. Nevertheless, how these phenotypic characteristics of males are translated at the behavioral level in females remains to be described.

In this study, we first identified that testosterone (T)-dependent changes in male feather coloration enhance female preferences for males in Japanese quail. We further found that this female mating preference is highly accelerated during the breeding season because the visual sensitivity of female birds coordinately increases with male attractiveness.

## Results

### Female birds prefer males with higher levels of circulating testosterone

**Figure 1:**
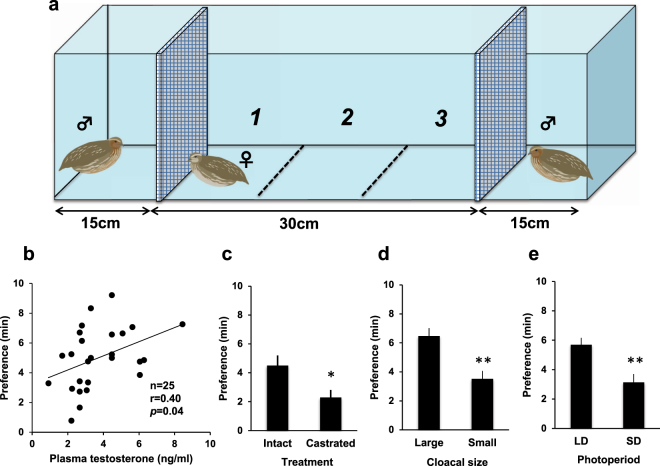
Female birds prefer males with higher levels of circulating testosterone. (**a**) Schematic drawing of the instrument used for mate preference tests. Each side of the instrument was separated by a net. (**b**) Relationship between male plasma testosterone levels and female mate choice. (**c**) Effects of castration on female preference (mean ± SEM, n = 15, *P = 0.03). (**d**) Female preference toward the male with large or small cloacal gland in size (mean ± SEM, n = 20, **P = 4.5 × 10^−4^). (**e**) Female preference for males maintained under long-day (LD: 14L-10D) or short-day (SD: 10L-14D) regimes (mean ± SEM, n = 12, **P = 1.1 × 10^−3^).

Since many testosterone-dependent traits have developed in the males of many animal taxa to attract females^16–18^, we examined whether the circulating levels of testosterone (T) in males affected female mate selection (Fig. 1a). We found a significant positive correlation between male T levels and female preference (Fig. 1b, r = 0.4, *P* = 0.04). These findings were corroborated by the observation that castration significantly reduced male attractiveness (Fig. 1c, *P* = 0.03). In addition, the size of the cloacal gland, which is a target organ of T in male quails^19^, shows a strong positive correlation with female preference (Fig. 1d, *p* = 4.5 × 10^−4^ and Supplementary Fig. S1a, r = 0.43, *P* = 0.03). Quail breed seasonally in response to changes in day length, which affects testes size. Since an increase in testes size results in higher levels of circulating T under long-day (LD) conditions^20^, we compared the attractiveness of males maintained under LD and short-day (SD) conditions. As expected, unlike the males maintained under SD conditions, the males maintained under LD conditions attracted significantly more females (Fig. 1e, *P* = 1.1 × 10^−3^). We performed microsatellite analysis to clarify the extent of genetic relatedness between males and females used in this study. In addition, we measured male body weight. Neither genetic relatedness between males and females (r = 0.07, *P* = 0.73), nor male body weight (r = 0.09, *P* = 0.63) were related to female mate preference in our experimental conditions (Supplementary Fig. S1b and c).

### Testosterone-dependent alteration of male feather coloration attracts females

**Figure 2:**
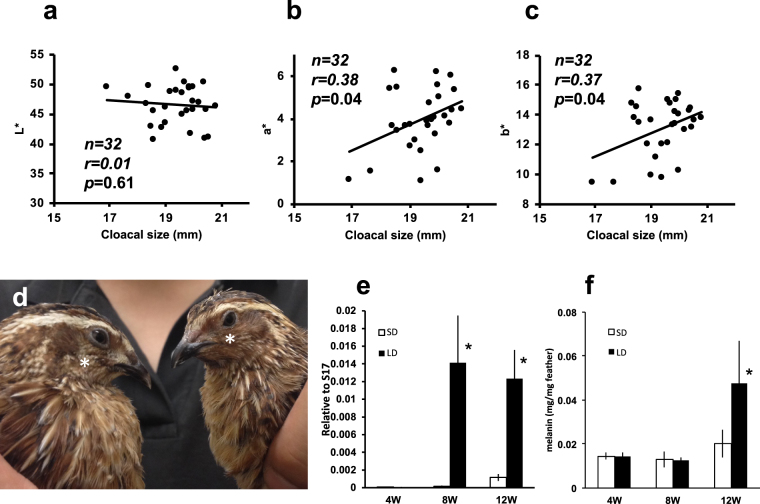
Attractive males possess more brightly colored cheek feathers. (**a**) Relationship between male cloacal size and colorimeter L* value (n = 32). (**b**) Relationship between male cloacal size and colorimeter a* value (n = 32). (**c**) Relationship between male cloacal size and colorimeter b* value (n = 32). (**d**) Appearance of intact male (right) and castrated male (left) at 13 weeks old. Asterisks indicate cheek area. (**e**) Tyrosinase gene expression in skin of males maintained for 4, 8 and 12 weeks under long-day (LD) and short-day (SD) conditions (mean ± SEM, n = 4, *P* = 0.79 at 4 W, *P* = 4.9 × 10^−2^ at 8 W and *P* = 3.4 × 10^−2^ at 12 W). (**f**) Melanin contents in the feathers of males maintained for 4, 8 and 12 weeks under long-day (LD) and short-day (SD) conditions (mean ± SEM, n = 5, *P* = 0.88 at 4 W, *P* = 0.88 at 8 W and *P* = 1.8 × 10^−2^ at 12 W).

As shown in Supplementary Fig. 2, castration reduced male attractiveness (Supplementary Fig. S2a, *P* = 0.02); however, male attractiveness could be recovered by T administration (Supplementary Fig. S2b). Importantly, measurements of male attractiveness over time showed that complete restoration of male attractiveness in castrated birds required continuous T administration for at least 4 weeks (Supplementary Fig. S2c). These findings showed that the traits that affect female mate preference may require a relatively long time to be fully expressed, and that the signal of male attractiveness may be maintained at higher level for the duration of the developmental period. Since the removal of male cheek feathers decreased male attractiveness (Supplementary Fig. S3, *P* = 5.2 × 10^−4^), we hypothesized that female attraction was due, at least in part, to changes in male cheek-feather coloration. To investigate whether there was a relationship between circulating T and male cheek-feather characteristics, we examined the color of male cheeks using a colorimeter. Because it is well known that the male cloacal gland is an external indicator of testicular development, thus resulting circulating T level, we measured cloacal size, and analyzed the link between male cheek patch coloration and T level. In colorimeter measurements, L* determines lightness, a* determines redness (+a*) or greenness (−a*), and b* determines yellowness (+b*) or blueness (−b*)^21^. Although the L* value was not related to the size of the cloacal gland (Fig. 2a, r = −0.1, *P* = 0.61), a statistically significant positive correlation was observed between cloacal gland size and the a* (r = 0.38, *P* = 0.04) and b* values (r = 0.38, *P* = 0.04) (Fig. 2b,c), that is, the feathers of attractive males expressed more red and yellow color. These results indicate that the cheek feathers of attractive males were more brilliantly colored than those of less attractive males. The appearance of intact and castrated birds (Fig. 2d) showed that the feathers of intact males appeared to be much browner than the feathers of castrated males. This prompted us to measure the expression of the tyrosinase gene, which is responsible for melanin synthesis in the skin^22^. As expected, compared to male birds maintained under SD conditions, tyrosinase expression increased significantly to a greater extent in sexually mature birds (>8 weeks of age) maintained under LD conditions (Fig. 2e, *P* = 4.9 × 10^−2^). Importantly, no significant difference was observed in the melanin content of feathers from SD and LD birds immediately after sexual maturation (8 weeks, *P* = 0.88); however, an increase in melanin content was observed after 12 weeks (Fig. 2f, *P* = 0.02). In addition, the recovery of attractiveness in castrated birds required 4 weeks of consecutive T injections (Supplementary Fig. S2c). These results indicate that full-term expression of male attractiveness in feather color requires several weeks after sexual maturation for a sufficient amount of melanin to accumulate. Indeed, the finding that the melanin content of cheek feathers was significantly correlated with both cloacal size (r = 0.62, *P* = 2.8 × 10^−3^) and female mate preference (r = 0.51, *P* = 0.03) corroborated this assumption (Supplementary Fig. S4). We therefore consider that the T-dependent color changes in male cheek feathers are an important trait for attracting females, and males must maintain this higher level throughout the breeding season by upregulating the tyrosinase expression and melanin deposition.

### Female mating preference solely works in breeding condition

**Figure 3:**
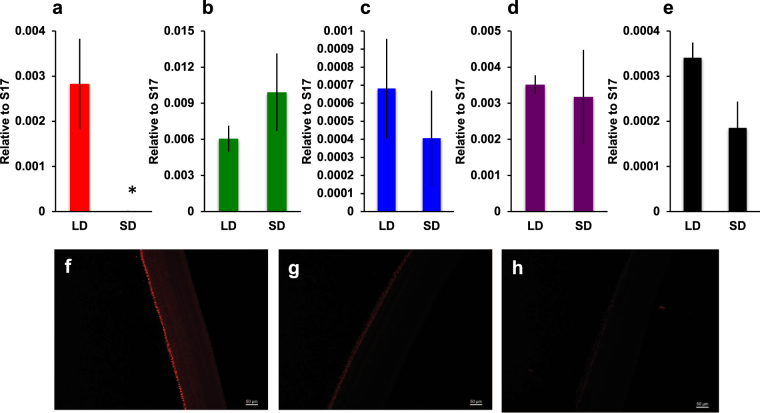
Expression of retinal photoreceptor genes in female birds. Real-time RT-PCR analysis of red- **(a)** green- **(b)** blue- **(c)** and violet- **(d)** cone sensitive opsin and rhodopsin **(e)** expression in females reared under long-day (LD) and short-day (SD) conditions (mean ± SEM, n = 4, *P* = 0.03 for red-opsin, *P* = 0.3 for green-opsin, *P* = 0.5 for blue-opsin, *P* = 0.8 for violet-opsin and *P* = 0.06 for rhodopsin). Immunohistochemical analysis of retina. **(f)** LD retina detected with anti-red/green opsin antibody; **(g)** SD retina detected with anti-red/green opsin antibody; **(h)** LD retina detected with normal mouse IgG. Bars = 50 μm.

**Figure 4:**
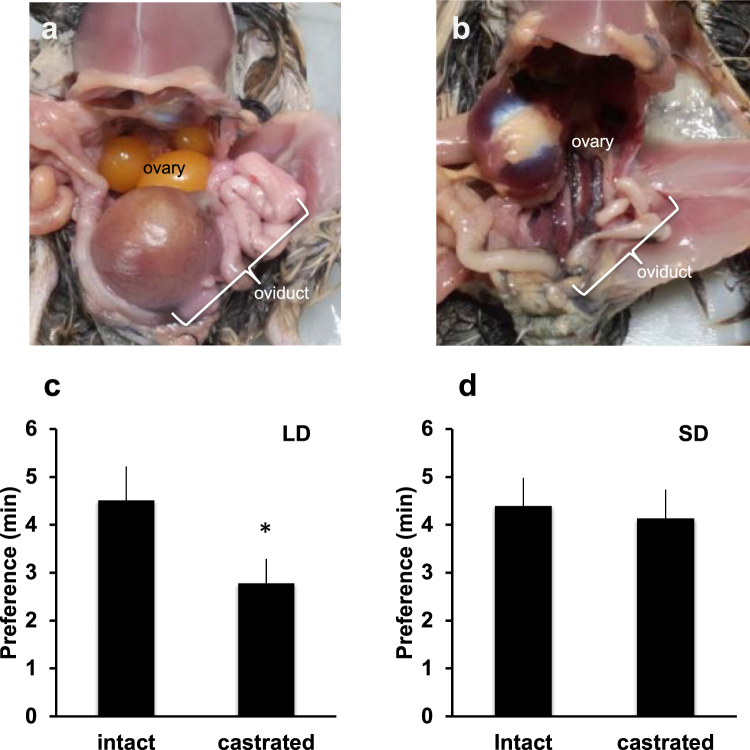
Female mating preference is accelerated under breeding conditions. Anatomy of female quail maintained under long-day (LD: 14L-10D) conditions **(a)** or short-day (SD: 10L-14D) conditions **(b)**. Mating preference tests with intact or castrated males performed with females maintained under LD **(c)** or SD **(d)** conditions (mean ± SEM, n = 15, *P = 1.7 × 10^−2^ for LD condition and *P* = 0.82 for SD condition).

How do females distinguish between attractive and unattractive males? To address this question, we performed mating preference tests using a light source that was covered with a sharp cut filter (SC64; cutoff <640 nm). We found that female birds were unable to distinguish between intact and castrated males (Supplementary Fig. S5), suggesting that female eyesight is critically important for mate preference. Next, we compared the expression level of the retinal photoreceptor gene in LD and SD females. Gene expression of the red-sensitive cone opsin was significantly lower in SD birds than in LD birds (Fig. 3a, *P* = 0.03). However, no such disparity was observed in the green-, blue- and violet-sensitive cone opsins (Fig. 3b–d) and the rhodopsin gene (Fig. 3e). Histological analysis did not reveal any obvious structural changes in the retinas of LD and SD birds (Supplementary Fig. S6). However, immunohistochemical analysis using anti-red/green opsin revealed that photoreceptor cell processes in LD birds contained more immunoreactive material than those in SD birds (Fig. 3f–h). When the female birds were maintained under SD conditions, they became reproductively inactive, and ovaries and oviducts decreased in size (Fig. 4a,b). In mate preference tests, we found that SD females also lost their ability to discriminate between intact and castrated males (Fig. 4c,d). These results support our hypothesis that female mate preference is highly stimulated when under LD conditions.

### Retinal photoreceptor expression is not under the influence of hormonal control

Retinal opsin expression is controlled by environmental stimuli, such as day length and environmental temperature^7^, and our results indicate the existence of a similar regulatory mechanism in the expression of the red-sensitive retinal cone opsin in Japanese quail. Since seasonal sex hormones influence opsin expression^23^, we administrated a variety of steroid hormones to SD females. Unexpectedly, none of these treatments affected red-sensitive cone opsin expression (Supplementary Fig. S7a). In birds, a recent study of Japanese quail revealed that LD-induced secretion of thyrotropin from the pars tuberalis of the pituitary gland causes local production of triiodothyronine (T_3_) in the mediobasal hypothalamus^24^. This local bioactive T_3_ regulates gonadotropin-releasing hormone, stimulating gonadotropin secretion and enhancing gonad development under LD conditions. We therefore performed intraventricular administration of thyrotropin (TSH), a regulator of seasonal breeding in birds, and evaluated the expression levels of red-sensitive cone opsin in the retina of SD birds. Unfortunately, this experiment failed to clarify the mechanism for upregulation of the red-sensitive cone opsin gene under LD conditions because TSH treatment had no effect on gene expression (Supplementary Fig. S7a). This failure was not due to problems with administration because both gonadal development (Supplementary Fig. S7b) circulating sex steroids (Supplementary Fig. S7c and d) increased markedly in TSH-treated animals. We also assessed the possibility that the retina itself recognizes the photoperiod. To test this hypothesis, LD-females were blindfolded with a black-colored rubber for 2 weeks. However, this treatment did not affect red opsin expression (Supplementary Fig. S8).

## Discussion

Given that sexual dimorphism is relatively subtle in the Japanese quail, it is not clear how females select mating partners in this species. Although we did not confirm whether males with higher circulating T without ornamental cheek patches were preferred by females, the results of this study clearly showed a relationship between male feather coloration and female mating preference. We also found that this feather coloration was exclusively T-dependent. Testosterone is an important mediator of reproductive behavior, including the establishment of territories, aggressive behavior and courtship displays or songs^25,26^. In addition, the development of secondary sexual ornaments, combs, wattles, plumes, and bill coloration are T-dependent^27,28^. Importantly, circulating T levels can reflect the nutritional condition of animals, because T levels decrease rapidly in animals experiencing food shortages^29^. Thus, in Japanese quail in which T-dependent cheek patch ornamentation attracts female birds provides support for the hypothesis that T-dependent traits reflect male quality and these traits affect female decision to mate. On the other hand the immunocompetence handicap hypothesis proposes that T has dual effects; it stimulates development of secondary sex characteristics, whereas it reduces immunocompetence, thus creating a physiological trade-off that influences, and is influenced by, parasite/pathogen attack. This balance is altered in accordance with the competing demands of the potential cost of infection versus that of increased reproductive success^30,31^. While it is currently unknown why females prefer higher T males in Japanese quail, the evidence suggests that aggressive male behavior, which is thought to be important to establish territories, is T-dependent^32^. In contrast, in the zebra finch (*Taeniopygia guttata*), Roberts *et al*. reported that female preference for males is not related to circulating T levels, but rather females preferred males from low corticosterone lines^33^. Although we are unable to give any conclusive statements, it is known that zebra finches are a monogamous species^34^ and female decisions over mating partners may be different from those of polygamous species such as Japanese quail. In this study, younger males (8 weeks) were selected as mating partners by females less frequently than older males (12 weeks). It is possible that older (and presumably more experienced) males carry genes that confer increased survival, compared to younger males, and that females then select those more experienced males as mating partners. Indeed, house wren (*Troglodytes aedon*) females were most likely to produce extra-pair offspring when paired with old males^35^. Although we did not compare attractiveness between yearling males and old males (more than one year old), one signal indicating male maturation is feather brightness and females may use this feather coloration as a marker for future survivability. Therefore, we think that full-term expression of cheek patch color advertises “full maturation” of the males.

Another significant finding is that this preference system is highly activated in the breeding season, and this is the first demonstration that visual sensitivity fluctuates seasonally in birds. Maney *et al*.^36^ suggested that in white‐throated sparrow (*Zonotrichia albicollis*) females, a song‐induced gene such as *zenk* (*egr‐1*) induction in the auditory system is selective for song only when plasma estradiol exceeds non‐breeding levels^36^. This finding indicated that the sensitivity of the auditory system also fluctuates in the breeding and non-breeding seasons and may be responsible for seasonally regulated sexual selection. Despite our best efforts to reveal the underlying mechanism, we were unable to elucidate how retinal red opsin is upregulated under LD conditions. It is not related to gonadal development because neither exogenous sex steroid administration nor central TSH infusion had an effect on upregulation of retinal red opsin. It is possible that another, as yet unknown mechanism exists, possibly regulated by day-length. In birds, the deep-brain photoreceptor opsin 5 was reported to be involved in gonad development under LD conditions through upregulating local TSH secretion by the pars tuberalis^37^. Our results suggest that this signaling pathway does not induce red-opsin expression in the retina, because upregulation of the target gene did not occur, despite obvious gonadal development and increased titers of circulating T and E_2_. These results imply that a photoreceptor other than opsin 5, which is responsible for the upregulation of red opsin, may exist. Future studies will attempt to elucidate the mechanisms of this putative photoreceptor and its signaling pathway.

In mice and humans, the major histocompatibility complex (MHC) is involved in mate selection, with mate odor used as a cue^38,39^. Similarly, although the precise mechanism is unknown, female sticklebacks prefer mating with partners that maximize the number of different MHC alleles of their offspring^40^. A number of studies have suggested that females favor sires that differ genetically from themselves. However, our results shown in Supplementary Fig. S1b suggest that female preference did not correlate with genetic relatedness between males and females, which was assessed by microsatellite analysis. (Supplementary Fig. S1b). In 1982, Bateson reported that the mating preference of female Japanese quail was related to their genetic dissimilarity and that they preferred their first cousins^41^. That study clearly demonstrated that genetic relatedness is one of the factors involved in the selection of a mating partner by females. In addition, Spottiswoode and Møller reported that inbreeding leads to a decrease in hatching success in nine wild bird species^42^, most probably due to the impairment of fertilization or embryonic death. The reason why we were unable to establish a correlation between genetic relatedness and female preference is unknown. However, one possible explanation is that we used domesticated quail and not wild birds. Such an inbred population sometimes may retrogress a trait that functions in wild counterparts (*i*.*e*. flight capability and incubating behavior etc.). Although we have no experimental support, our results indicate the possibility that a T-mediated mate selection system may be more primitive/fundamental than a system based on genetic dissimilarity, because the T-mediated mate selection system works in domestic birds such as Japanese quail.

Taken together, we have demonstrated that female mating preference in the Japanese quail is stimulated by male feather coloration in a T-dependent manner and that female birds develop a keen sense for this coloration due to upregulation of retinal red-sensitive opsin under breeding conditions. While we are curious about the mechanism underlying the upregulation of red opsin under LD conditions, further research is required to clarify this point. It is expected that elucidation of this mechanism will open new avenues in avian reproductive physiology, especially in the fields of sexual selection and seasonal breeding activity.

## Methods

### Animal rearing and maintenance

Five to 15 week-old Japanese quail (Quail Cosmos Co., Toyohashi Japan) were maintained individually at 20–25 °C under a photoperiod of 14 L:10D (lights on at 05:00) for long-day conditions. For short-day conditions, birds were maintained under a photoperiod of 10 L:14D (lights on at 07:00). All birds were given *ad libitum* access to water and a commercial diet (Hokkaiya Co, Aichi, Japan). Castration was performed under deep anesthesia by isoflurane inhalation (Wako pure chemicals, Osaka Japan). The cheek area feathers in males were removed using forceps. The size of the cloacal gland was measured using calipers. Blood sampling was performed from the wing-vein and plasma testosterone and estradiol-17β were measured using an ELISA kit (Cayman, Ann Arbor, MI). In the blindfold treatment, we covered the left eyes of females by rubber plunger of 2.5 ml disposable syringe (TERUMO Corp., Tokyo, Japan), which was fixed by adhesive in order to shut out the transmitted light. Retinas and skin samples of female birds were isolated after cervical dislocation at 2 h after dawn. All experimental procedures for the care and use of animals were carried out in accordance with the approved guidelines of the Animal Care Committees of Shizuoka University (Approval number: 28–13, 29A-11).

### Analysis of genetic relatedness

We performed microsatellite analysis to clarify the extent of genetic relatedness among the birds used in this study. DNA samples were isolated from blood and used for PCR amplification. Primers were prepared for 19 microsatellite loci (GUJ0005, 11, 13, 14, 17, 21, 23, 26, 28, 40, 41, 44, 49, 54, 55, 65, 69, 71, 84, 85)^43^. The forward primers were labeled at the 5′ end with 6-FAM, 5-HEX or NED. Multiplex PCR was carried out with two or three primer pairs in a total volume of 12.5 μL (1 μL of genomic DNA, 1× PCR buffer, 0.2 mM dNTPs, 1.5 mM MgSO_4_, 0.1 μM each of primers and 0.25 U of KOD -Plus- Neo DNA polymerase (TOYOBO, Osaka, Japan)). The PCR profile consisted of 94 °C for 2 min, followed by 30 cycles of 98 °C for 10 s, 55–62 °C for 30 s and 68 °C for 30 s. Annealing temperatures for each reaction were chosen according to a previous report^43^. Then, 0.5 μL of PCR product was mixed with 0.5 μL of GeneScan™ 500 LIZ^®^ Size Standard (Applied Biosystems, Foster City, CA) and 9 μL of formamide, and denatured at 95 °C for 5 min. This mixture was used to determine the number of repeats in the microsatellites using a 3500 × l Genetic Analyzer (Applied Biosystems). The datasets were then analyzed for allele calling using GeneMapper 5.0 (Applied Biosystems). Genetic relationships were calculated based on the 19 microsatellite genotypes using GENEPOP version 4.2^44,45^.

### Hormone treatment

Estradiol-17β, testosterone and progesterone were obtained from Sigma Japan (Tokyo, Japan). The hormones were dissolved in sesame oil (1 mg/ml) and *subcutaneously* injected in the back at a dose of 0.1 mg/100 g body weight. The same volume of sesame oil was injected as a vehicle control. Hormone injections (in Supplementary Figs S2 and S7a) were performed twice a week.

### Intraventricular administration

Females were deeply anesthetized by isoflurane inhalation and placed in a stereotaxic instrument (David Kopf Instruments, Tujunga, CA). A guide cannula (AG-8; Eicom, San Diego, CA) was implanted stereotaxically into the cerebral ventricle (3.0 mm anterior, 0 mm lateral from the Y-point and 7.0 mm deep from the surface of the dura matter) and fixed to the skull with dental cement. An obturator was inserted into the guide cannula until experiments began. 3 days after surgery, we injected bovine TSH (T8931, Sigma Japan) or PBS through a guide cannula and measured the resulting changes in target gene expression. Injections were given every 2 days and continued for 2 weeks. We used bovine TSH because avian TSH was not available, and bovine TSH is known to activate the avian TSH receptor^46^.

### Mating preference tests

The testing apparatus used to assess mating preference consisted of a plastic box (60 cm × 30 cm × 30 cm) with two boundaries. Each boundary was positioned 15 cm away from the long edge of the box and comprised wire netting (Fig. 1a). The apparatus was lit from the top by two 20 W lamps. The center area of the apparatus was divided into 3 parts (blocks 1–3), each of which was 10 cm wide. To begin the mating preference test, the stimulus males were each placed on the one side of the focal female, which was placed in a holding box at the center of the apparatus. After 30 s, the holding box was removed, allowing the female to move around the enclosure. The first 1 min was disregarded as acclimation, and preference data were recorded for the next 5 min. We recorded the time that the female spent on each side (block 1 or 3) and assumed that she spent more time near the preferred male. After 5 min, the positions of the stimulus males were reversed (i.e. the male on one side was placed on the other side and vice versa), and another 5 min recording was performed. In total, 10 min of testing was performed and the time the female spent on each side of the stimulus male was calculated. To investigate if female eyesight is important for mate preference, mating preference tests were performed under lamps covered with a sharp cut filter (SC64; Fuji Film Co., Tokyo, Japan; cutoff < 640 nm).

### Evaluation of feather color by colorimeter

The cheek area feathers of males were removed and placed on a sheet of black paper (3 cm^2^). The absolute values of L*, a* and b* were then measured using a colorimeter (CR-400, Konica-Minolta, Tokyo, Japan). The Lab color space describes mathematically all perceivable colors in the three dimensions L for lightness and a and b for the color components green–red and blue–yellow, respectively. In colorimeter measurement, L* determines lightness, a* determines redness (+a*) or greenness (−a*), and b* determines yellowness (+b*) or blueness (−b*)^21^. L* value ranges between 0 and 100, a* value ranges between −90 and 70, and b* value ranges between −80 and 100.

### Measurement of eumelanin in cheek feathers

The eumelanin content in cheek feathers was determined as described previously^47^. Briefly, a 15–20 mg feather was cut into small pieces and homogenized in 1N H_2_SO_4_ (5 mg feather in 1 ml). Then, 3 ml of feather homogenate was added to the tube and oxidized by adding drops of 3% KMnO_4_ with constant mixing. After 10 min of oxidation, excess KMnO_4_ was removed by adding 10% Na_2_SO_3_, and the resulting mixture was extracted by ether. The organic phase was collected and evaporated to dryness and the residue was dissolved in water. The oxidized product of eumelanin, pyrrole-2,3,5-tricarboxylic acid (PTCA), was analyzed by HPLC (LC-2000, JASCO) using a C-18 reverse phase column (Wakosil-II 5C18 RS, ∅3.0 mm × 150 mm, Wako Pure Chemicals, Osaka, Japan). The sample was eluted with 0.1 M potassium phosphate buffer (pH 2.1) containing 4% methanol at a flow rate of 0.35 ml/min at 55 °C, and the absorbance at 254 nm was recorded. The peak eluted at 8.9 min was determined to be PTCA using a standard curve made using PTCA derived from known amounts of synthetic melanin purchased from Sigma Japan.

### Real-time RT-PCR

Total RNA was isolated from the dissected tissue (retina of right eye and skin from the cheek area) using a RNAiso kit (Takara Biomedical, Otsu, Japan) according to the manufacturer’s instructions. Aliquots (0.5 μg) were reverse transcribed at 37 °C for 15 min with ReverTra Ace qPCR kit (TOYOBO), and the reaction product was subjected to real-time PCR according to the instructions for the Light Cycler Nano System with the FastStart Essential DNA Green Master (Roche Applied Science, Penzberg, Germany). Briefly, following a denaturing step at 95 °C for 10 s, PCR was performed using a thermal protocol consisting of 95 °C for 20 s, 57.6 °C for 20 s and 72 °C for 20 s for red-sensitive cone opsin, 95 °C for 20 s, 55.4 °C for 20 s and 72 °C for 20 s for green-, blue- and violet-cone sensitive opsins and tyrosinase, and 95 °C for 20 s, 60.0 °C for 20 s and 72 °C for 20 s for rhodopsin and S17 in 20 μl buffer containing 0.2 μM of each primer. The sense and antisense primers used for red-, green-, blue- and violet-cone sensitive cone opsin, rhodopsin and tyrosinase amplification were 5′-TGCTCTGCTACCTGCAAGTCT-3′ and 5′-GGGGTTATAGATCGTTGCTGAC -3′, respectively (GenBank Accession number: XM_015850819), 5′-ACAACCCCGACTACCACAAC -3′ and 5′-TCCCTTGTTGGTGAAGATCC-3′, respectively (GenBank Accession number: XM_015885397), 5′-CTCAGCCCCTTCTTAGTCCC-3′ and 5′-GGGTCCCAAAGCGAAATACA-3′, respectively (GenBank Accession number: NM_205517), 5′-CCTCGGACGACGACTTCTAC-3′ and 5′-CCGAGATTGTTGACCAGGAT-3′, respectively (GenBank Accession number: XM_015851133), 5′-CTTTTTGGCATGCTCTGTGA-3′ and 5′-GCCCACAGTATGGCTGAGAT-3′, respectively (GenBank Accession number: EU737202), 5′-CTTACTGCTGGCCATCCTTC-3′ and 5′-TGGGGATGTTCTTTGCTAGG-3′, respectively (GenBank Accession number: NM_001323239). For normalization of the data, we amplified the *S17* gene (GenBank Accession number: AY232491, sense primer; 5′-CCAGACACCAAGGAGATGCT-3′, antisense primer; 5′-GCCTCGTGGTGTTTTGAAGT-3′) using the same cycle conditions as for the target genes. To normalize the data, ΔCT was calculated for each sample by subtracting the CT value for *S17* from the CT value for the target gene and 2^−ΔCT^ was calculated. The results were expressed as the target gene mRNA/*S17* mRNA ratio.

### Immunohistochemical detection of photoreceptors in the retina

The female birds were sacrificed, and the left eyeball was removed. The retina was isolated and fixed in Bouin’s fixative, and the tissue was embedded in paraffin. The immunohistochemical techniques were performed as described previously^48^, and rabbit anti-human opsin antibody (AB5405, Merck Inc., Kenilworth, NJ) and Alexa Fluor 514 conjugated anti-rabbit IgG (ThermoFisher, Waltham, MA) were used as primary and secondary antibodies, respectively. The immunolabeled sections were examined under a fluorescence microscope (BX51; Olympus, Tokyo, Japan). Some paraffin sections were stained with H&E to observe structural differences of retina between long-day and short-day conditions.

### Statistical analysis

All data were analysed by first using Kolmogorov–Smirnov test using R version 3.3.3 (The R Foundation for Statistical Computing Platform, https://cran.r-project.org/bin/macosx/) to determine if the data were normally distributed. All the data reported here were normally distributed and the data are shown as mean ± SEM. F-tests were used to determine variance and the data with a normal distribution were analyzed by a Student’s t-test between two groups. Differences were considered significant at *P* < 0.05. We used a two-sided Pearson’s correlation test to measure the strength of the relationship between the two parameters.

## Electronic supplementary material

Supplementary information

## References

[1] Andersson, M. *Sexual Selection* (Princeton University Press, 1994).

[2] Dale J, Dey CJ, Delhey K, Kempenaers B, Valcu M (2015). The effects of life history and sexual selection on male and femlae plumage colouration. Nature.

[3] Kvarnemo C, Simmons LW (2013). Polyandry as a mediator of sexual selection before and after mating. Phil. Trans. R. Soc. B.

[4] Bateman AJ (1948). Intra-sexual selection in Drosophila. Heredity (Edinb).

[5] Chang H (2017). A pheromone antagonist regulates optimal mating time in the moth *Helicoverpa armigera*. Curr. Biol..

[6] Houde AE (1987). Mate choice based upon naturally occurring color-pattern variation in a guppy population. Evolution (N. Y).

[7] Shimmura T (2017). Dynamic plasticity in phototransduction regulates seasonal changes in color perception. Nat. Commun..

[8] Okuyama T (2014). A neural mechanism underlying mating preferences for familiar individuals in medaka fish. Science.

[9] Stieb SM, Carleton KL, Cortesi F, Marshall NJ, Salzburger W (2016). Depth-dependent plasticity in opsin gene expression varies between damselfish (Pomacentridae) species. Mol. Ecol..

[10] Shao YT (2014). Androgens increase lws opsin expression and red sensitivity in male three-spined Sticklebacks. PLoS One.

[11] Matos-Cruz V (2011). Unexpected diversity and photoperiod dependence of the zebrafish melanopsin system. PLoS One.

[12] Welbourne LE, Morland AB, Wade AR (2015). Human colour perception changes between seasons. Curr. Biol..

[13] Hamilton WD, Zuk M (1982). Heritable true fitness and bright birds: a role for parasites?. Science.

[14] Hill GE (1993). Male mate choice and the evolution of female plumage coloration in the house finch. Evolution (N. Y).

[15] Zuk M, Thornhill R, Ligon JD, Johnson K (1990). Parasites and mate choice in red jungle fowl. Am. Zool..

[16] Henly CL, Nunez AA, Clements LG (2011). Hormone of choice: The neuroendocrinology of partner preference in animals. Front. Neurosci..

[17] Longpre KM, Katz LS (2011). Estrous female goats use testosterone-dependent cues to assess mates. Horm. Behav..

[18] Taylor GT, Haller J, Regan D (1982). Female rats prefer an area vocated by a high testosterone male. Physiol. Behav..

[19] Nagra CL, Meyer RK, Bilstad N (1959). Cloacal glands in Japanese quail (*Coturnix coturnix japonica*): Histogenesis and response to sex steroids. Anat. Rec..

[20] Follett BK, Maung SL (1978). Rate of testicular maturation, in relation to gonadotrophin and testosterone levels, in quail exposed to various artificial photoperiods and to natural daylengths. J. Endocrinol..

[21] Yuan J, Brewer JD, Monaco E, Davis E (2007). Defining a natural tooth color space based on a 3dimensional shade system. J. Prosthet. Dent..

[22] Wakamatsu K, Ito S, Rees JL (2002). The usefulness of 4-amino-3-hydroxyphenylalanine as a specific marker of pheomelanin. Pigment Cell Res..

[23] Hope AJ, Partridge JC, Hayes PK (1998). Switch in rod opsin gene expression in the European eel, *Anguilla anguilla* (L.). Proc. R. Soc. B Biol. Sci..

[24] Yamamura T, Hirunagi K, Ebihara S, Yoshimura T (2004). Seasonal morphological changes in the neuro-glial interaction between gonadotropin-releasing hormone nerve terminals and glial endfeet in Japanese quail. Endocrinology.

[25] Lofts, B. & Murton, R. K. Reproduction in birds in *Avian Endocrinology*, (ed. Farner, D.S. & King, J.R.) 1–107 (Academic Press, 1973).

[26] Wingfield JC, Hegner RE, Dufty AM, Ball GF (1990). The “challenge hypothesis”: theoretical implications for patterns of testosterone secretion, mating systems, and breeding strategies. Am. Nat..

[27] Bentley, P. J. *Comparative Vertebrate Endocrinology* (Cambridge University Press, 1976).

[28] Johnson, A. L. Reproduction in the male. In *Avian Phyiology*, *Fouth Edition*, (ed. Sturkie, P. D.) 432–451 (Spriger, 1986).

[29] Wingfield, J. C. Changes in reproductive function of free-living birds in direct response to environmental perturbations in *Processing of Environmental Information in Vertebrates*, (ed. Stetson M. H.) 121–148 (Springer, 1988).

[30] Folstad I, Karter AJ (1992). Parasites, bright males, and the immunocompetence handicap. Am. Nat..

[31] Roberts ML, Buchanan KL, Evans MR, Marin RH, Satterlee DG (2009). The effects of testosterone on immune function in quail selected for divergent plasma corticosterone response. J. Exp. Biol..

[32] Ubuka T (2014). Hypothalamic inhibition of socio-sexual behaviour by increasing neuroestrogen synthesis. Nat. Commun..

[33] Roberts ML, Buchanan KL, Bennett ATD, Evans MR (2007). Mate choice in zebra finches: does corticosterone play a role?. Anim. Behav..

[34] Zann, R. A. The zebra finch: *A synthesis of field and laboratory studies*. (Oxford Univeristy Press, 1996).

[35] Bowers EK (2015). Increased extra-pair paternity in broods of aging males and enhanced recruitment of extra-pair young in a migratory bird. Evolution.

[36] Maney DL, Cho E, Goode CT (2006). Estrogen-dependent selectivity of genomic responses to birdsong. Eur. J. Neurosci..

[37] Nakane Y (2010). A mammalian neural tissue opsin (Opsin 5) is a deep brain photoreceptor in birds. Proc. Natl. Acad. Sci..

[38] Wedekind C, Seebeck T, Bettens F, Paepke AJ (1995). MHC-dependent mate preferences in humans. Proc. R. Soc. London B Biol. Sci..

[39] Chaix R, Cao C, Donnelly P (2008). Is mate choice in humans MHC-dependent?. PLoS Genet..

[40] Reusch TBH, Häberli MA, Aeschlimann PB, Milinski M (2001). Female sticklebacks count alleles in a strategy of sexual selection explaining MHC polymorphism. Nature.

[41] Bateson P (1982). Preferences for cousins in Japanese quail. Nature.

[42] Spottiswoode C, Møller AP (2004). Genetic similarity and hatching success in birds. Proc. R. Soc. B Biol. Sci..

[43] Kayang BB (2004). A first-generation microsatellite linkage map of the Japanese quail. Anim. Genet..

[44] Raymond M, Rousset F (1995). GENEPOP (version 1.2): population genetics software for exact tests and ecumenicism. J. Hered..

[45] Rousset F (2008). Genepop'007: a complete reimplementation of the Genepop software for Windows and Linux. Mol. Ecol. Resources.

[46] Grommen SV (2006). D. Molecular cloning, tissue distribution, and ontogenic thyroidal expression of the chicken thyrotropin receptor. Endocrinology.

[47] Haase E, Ito S, Sell A, Wakamatsu K (1992). Melanin concentrations in feathers from wild and domestic pigeons. J. Hered..

[48] Sasanami T (2002). Secretion of egg envelope protein ZPC after C-terminal proteolytic processing in quail granulosa cells. Eur. J. Biochem..

