# Supplementary material for: Female Japanese quail visually differentiate testosterone-dependent male attractiveness for mating preferences
Source: Sci Rep. 2018 Jul 3;8:10012. doi: 10.1038/s41598-018-28368-z (PMC6030125; doi:10.1038/s41598-018-28368-z)
Supplement: Supplementary file 1 — Supplementary information [file 41598_2018_28368_MOESM1_ESM.docx]

Female Japanese quail visually differentiate testosterone-dependent male attractiveness for mating preferences

Gen Hiyama^1, †^, Shusei Mizushima^2^, Mei Matsuzaki^1^, Yasuko Tobari^3^, Jae-Hoon Choi^1^, Takashi Ono^4^, Masaoki Tsudzuki^4^, Satoshi Makino^5^, Gen Tamiya^5,6^, Naoki Tsukahara^7^, Shoei Sugita^8^ and Tomohiro Sasanami^1*^

^1^Department of Applied Biological Chemistry, Faculty of Agriculture, Shizuoka University, 836 Ohya, Shizuoka, Shizuoka 422-8529, Japan.

^2^Department of Biological Sciences, Faculty of Science, Hokkaido University, Kita 10 Nishi 8, Kita-ku, Sapporo, Hokkaido 060-0810, Japan.

^3^Laboratory of Animal Genetics and Breeding, School of Veterinary Medicine, Azabu University, Fuchinobe 1-17-71, 252-5201, Japan.

^4^Laboratory of Animal Breeding and Genetics, Graduate School of Biosphere Science, Hiroshima University, Higashi-Hiroshima 739-8528, Japan.

^5^Tohoku Medical Megabank Organization, Tohoku University, 2-1 Seiryo-machi, Aoba-ku, Sendai, Miyagi 980-8575, Japan

^6^RIKEN Center for Advanced Intelligence Project, 1-4-1 Nihonbashi, Chuo-ku, Tokyo 103-0027, Japan

^7^Utsunomiya-ventures #3, Tochigi Prefecture Industrial Center, 3-1-4, Chuo, Utsunomiya-shi, Tochigi, 320-0806, Japan

^8^Faculty of Agriculture, Utsunomiya University, Utsunomiya, Tochigi 321-8505, Japan.

†Current address. Medical-Industrial Translational Research Center, Fukushima Medical University, 11-25 Sakaemachi, Fukushima 960-8031, Japan.

*Correspondence to: Tomohiro Sasanami, Department of Applied Biological Chemistry, Faculty of Agriculture, Shizuoka University, 836 Ohya, Shizuoka, Shizuoka 422-8529, Japan. Email: atsasan@shizuoka.ac.jp

**Supplementary Information**

Supplementary Figure S1: Female prefer neither genetically dissimilar males nor large body sized males

Supplementary Figure S2: Effect of testosterone treatment on recovery of female mating preference in castrated males.

Supplementary Figure S3: Effect of cheek feather removal on male attractiveness.

Supplementary Figure S4: Feather melanin contents in males.

Supplementary Figure S5: Female birds visually differentiate preferred males

Supplementary Figure S6: Histochemical observations of female retinas.

Supplementary Figure S7: Red-sensitive cone opsin expression is not related with gonadal development.

Supplementary Figure S8: Effects of blindfold on expression of red-sensitive cone opsin.

**
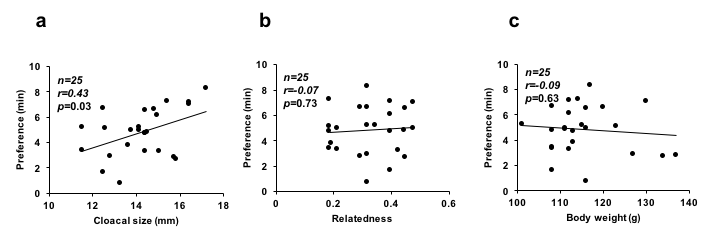
**

**Supplementary Figure S1: Female prefer neither genetically dissimilar males nor large body sized males.**

Relationship between female preference and size of male cloacal gland **(a)**, relatedness **(b)**, and male body weight **(c)** (n=25).

**
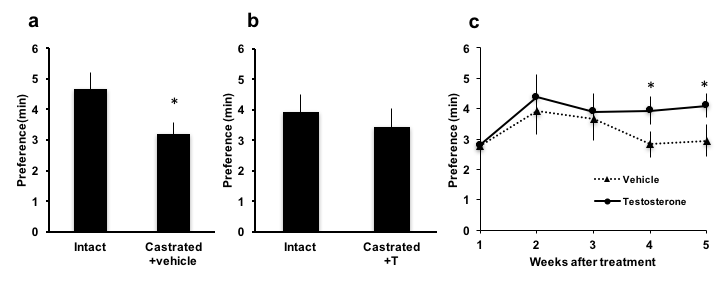
**

**Supplementary Figure S2: Effect of testosterone treatment on recovery of female mating preference in castrated males.**

Castrated males received vehicle **(a)** or testosterone **(b)** injection for 5 weeks and mate preference tests were performed (mean ± SEM, n=15-16, **P*=1.7 x 10-2 for vehicle treatment and *P*=0.69 for testosterone treatment). **c,** Time course studies on effects of testosterone treatment. Mate preference tests were performed every week (mean ± SEM, n=10-15, *P*=0.49 at 1W, *P*=0.68 at 2W, *P*=0.81 at 3W, *P*=0.05 at 4W and *P*=0.04 for 5W).

**
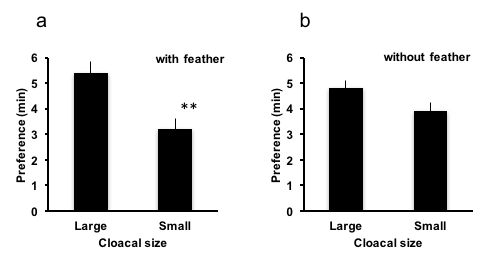
**

**Supplementary Figure S3: Effect of cheek feather removal on male attractiveness.**

**a,** Intact males were divided into two groups according to size of cloacal gland, and mate preference tests were performed (mean ± SEM, n=25, ***P*=5.2 x 10^-4^). **b,** Cheek feathers of males were removed, and mate preference tests were performed (mean ± SEM, n=17, *P*=0.06).

**
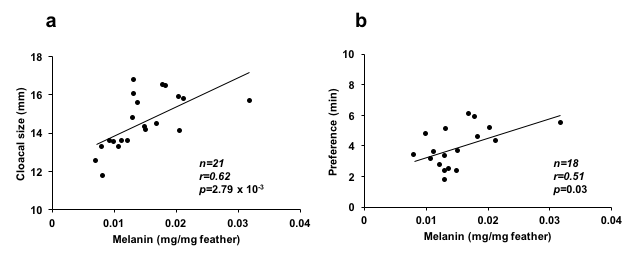
**

**Supplementary Figure S4: Feather melanin contents in males.**

**a,** Relationship between melanin contents in cheek feathers and size of cloacal gland (n=21). **b,** Relationship between melanin contents in cheek feathers and female mate preference (n=18).

**
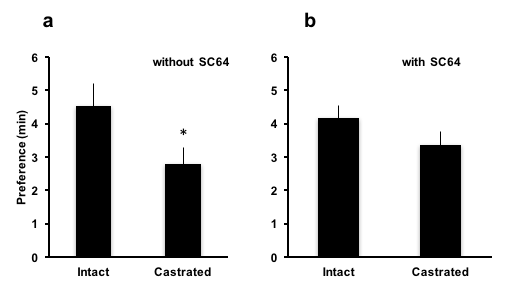
**

**Supplementary Figure S5: Female birds visually differentiate preferred males.**

**a,** Mating preference tests using intact or castrated males performed under standard light conditions (mean ± SEM, n=15, **P*=0.03). **b,** Mating preference tests in **(a)** performed under light source covered with sharp cut filter (cutoff : <640 nm) (mean ± SEM, n=27, *P*=0.07).

**
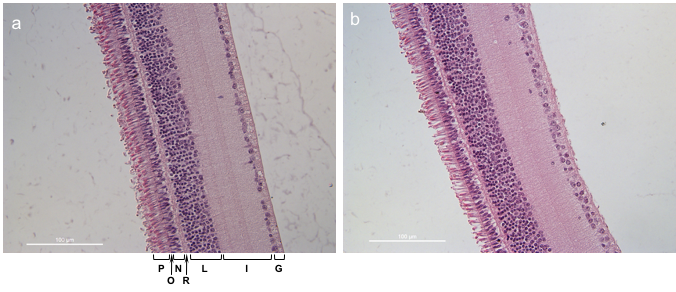
**

**Supplementary Figure S6: Histochemical observations of female retinas.**

**a,** HE stained retina of LD female. **b,** HE stained retina of SD female. P, photoreceptor cell processes; O, outer limiting membrane; N, external nuclear layer; R, external plexiform layer; L, internal nuclear layer; internal plexiform layer; G, ganglion cell layer. Scale bars = 100 μm.

**
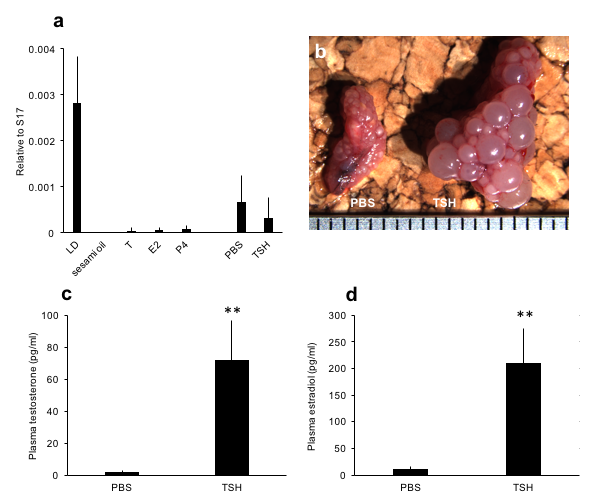
**

**Supplementary Figure S7: Red-sensitive cone opsin expression is not related with gonadal development.**

**a,** Effects of various steroid hormone injection (testosterone, estradiol-17β or progesterone) and intraventricular administration of TSH on the expression of retinal red-sensitive cone opsin (mean ± SEM, n= 4-8, *P*=1.8 x 10^-3^ for LD vs sesame oil, *P*=7.7 x 10^-3^ for LD vs T, *P*=8.1 x 10^-3^ for LD vs E_2_, *P*=8.5 x 10^-3^ for LD vs P_4_, and *P*=0.66 for PBS vs TSH). **b,** Appearance of ovary of PBS or TSH treated females. **c,** Plasma testosterone level in PBS or TSH treated females (mean ± SEM, n= 3, ***P*=4.9 x 10^-2^). **d,** Plasma estradiol-17β level in PBS or thyrotropin treated females (mean ± SEM, n= 3, ***P*=3.6 x 10^-2^).

**
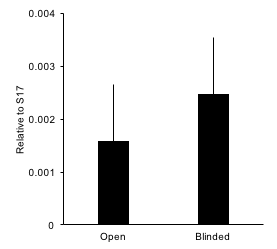
**

**Supplementary Figure S8: Effects of blindfold on expression of red-sensitive cone opsin.**

Females maintained under LD conditions (14L-10D) had their left eye blindfolded (blinded) and their right eye was retained intact (open) for 2 weeks, and retinal red-sensitive opsin expression was evaluated. Values are mean ± SEM (n=7, *P*=0.57).
